# Fusion Ranging Method of Monocular Camera and Millimeter-Wave Radar Based on Improved Extended Kalman Filtering

**DOI:** 10.3390/s25103045

**Published:** 2025-05-12

**Authors:** Ye Chen, Qirui Cui, Shungeng Wang

**Affiliations:** School of Engineer, Huzhou University, Huzhou 313000, China; 2023388619@stu.zjhu.edu.cn (Q.C.); 2023388606@stu.zjhu.edu.cn (S.W.)

**Keywords:** monocular ranging, millimeter-wave radar, multi-sensor fusion, extended Kalman filter

## Abstract

To address the limitations of single-sensor systems in environmental perception, such as the difficulty in comprehensively capturing complex environmental information and insufficient detection accuracy and robustness in dynamic environments, this study proposes a distance measurement method based on the fusion of millimeter-wave (MMW) radar and monocular camera. Initially, a monocular ranging model was constructed based on object detection algorithms. Subsequently, the pixel-distance joint dual-constraint matching algorithm is employed to accomplish cross-modal matching between the MMW radar and the monocular camera. Furthermore, an adaptive fuzzy extended Kalman filter (AFEKF) algorithm was established to fuse the ranging data acquired from the monocular camera and MMW radar. Experimental results demonstrate that the AFEKF algorithm achieved an average root mean square error (RMSE) of 0.2131 m across 15 test datasets. Compared to the raw MMW radar data, inverse variance weighting (IVW) filtering, and traditional extended Kalman filter (EKF), the AFEKF algorithm improved the average RMSE by 10.54%, 11.10%, and 22.57%, respectively. The AFEKF algorithm improves the extended Kalman filter by integrating an adaptive fuzzy mechanism, providing a reliable and effective solution for enhancing localization accuracy and system stability.

## 1. Introduction

### 1.1. Research Background and Motivation

With the rapid advancement of advanced driver assistance systems (ADASs) and intelligent robotic platforms, accurate environmental perception has become a core requirement [1]. In complex and dynamic scenarios, real-time and reliable distance measurement serves not only as a fundamental support for tasks such as obstacle detection, collision warning, and autonomous navigation but also as a key factor in ensuring system safety and enhancing operational efficiency [2,3]. Whether it involves precise localization of surrounding objects for autonomous vehicles or obstacle avoidance in unstructured environments for robots [4], high-precision distance measurement remains essential for enabling autonomous decision-making and intelligent control.

As the core of environmental perception, sensor performance directly impacts the accuracy of perception. Consequently, extensive research has been conducted on devices such as millimeter-wave radar, LiDAR, and cameras [5,6,7]. Cameras have been widely adopted in detection tasks due to their rich texture information and low cost. However, inherent limitations in imaging principles and physical characteristics hinder their ability to perceive depth. Some studies have attempted to achieve distance estimation by defining geometric relationships and incorporating spatial priors [8,9]; nevertheless, such methods are often constrained by specific scenarios. With the rapid development of deep learning, end-to-end visual ranging approaches based on image features and distance mapping have significantly improved robustness [10,11,12,13,14]. However, their accuracy depends heavily on algorithmic precision and large-scale, high-quality training datasets, leading to substantial errors in complex environments. Moreover, these methods are susceptible to variations in lighting, weather, and environmental complexity [15]. In contrast, millimeter-wave radar is less affected by adverse weather conditions such as rain, snow, or fog, offering higher environmental adaptability, and it has been widely used in automotive applications [16]. Some researchers have leveraged deep learning techniques to process millimeter-wave radar point cloud data for road target detection and tracking [17,18,19]. However, the sparse and low-resolution nature of radar point clouds still limits their application in high-precision perception tasks.

### 1.2. Related Work

Due to the inherent limitations of individual sensors under varying environmental conditions, it is challenging for a single modality to meet the demands of all-weather and high-precision detection [20]. Cameras can provide rich texture information and enable distance estimation; however, their ranging accuracy is highly sensitive to environmental changes. Millimeter-wave radars, in contrast, offer stable distance measurements but suffer from low resolution. These two modalities exhibit strong complementarity in their perception capabilities. Therefore, the multi-modal fusion of cameras and millimeter-wave radar is considered a key strategy for overcoming current perception bottlenecks [21,22]. In [23], an improved radar tracking and visual detection algorithm was proposed for forward collision warning, using decision-level fusion to significantly reduce false positives and missed detections in complex environments. Paper [24] employed global nearest-neighbor data association combined with extended Kalman filtering to leverage the ranging and speed detection strengths of radar along with the target recognition capabilities of cameras. The method achieved an obstacle detection accuracy of 86.18% on a tractor-based experimental platform. In [25], a loop closure detection system was developed by combining features extracted via convolutional neural networks with linear characteristics, using millimeter-wave radar to measure train speed and thereby enabling precise localization through loop closure results. In [26], a sensor association method which enhanced the accuracy and robustness of pedestrian tracking was proposed based on back-projection and undirected graph optimization for radar–camera fusion. Paper [27] projected radar point cloud data onto image space to guide visual feature fusion, demonstrating superior performance on the NuScenes dataset.

In summary, the fusion of camera and millimeter-wave radar technologies has significantly progressed. However, existing studies have primarily focused on optimizing fusion strategies for object detection, tracking, and localization tasks, while the enhancement of multi-modal ranging accuracy in dynamic environments remains under-explored.

Some researchers have applied traditional fusion algorithms, such as Kalman filtering [28,29,30], to fuse sensor measurements. These approaches typically assume fixed sensor measurement errors and tend to yield satisfactory results in static scenarios [31,32]. In practice, however, targets are usually in motion, and sensor performance often varies with target dynamics. Consequently, the associated measurement noise becomes non-stationary, making it difficult for conventional methods to maintain high accuracy in dynamic environments.

### 1.3. Our Contributions

To address these challenges, this paper proposes a distance estimation method for dynamic scenarios based on the fusion of millimeter-wave radar and monocular camera data. The main contributions are as follows:

- A monocular ranging model is constructed based on a target detection algorithm after achieving spatio-temporal calibration between the radar and camera. A pixel–distance dual-constraint matching algorithm is employed to achieve effective cross-modal target association between the two sensors.
- An adaptive fuzzy extended Kalman filter (AFEKF) algorithm is proposed. This algorithm dynamically updates the measurement error covariance matrix based on the target’s distance variation and adaptively adjusts the sensor fusion weights in each frame according to the variations in sensor measurements. The effectiveness of the proposed algorithm in improving fusion accuracy for dynamic target ranging is validated through experiments.

The structure of this paper is organized as follows: Section 2 introduces the spatio-temporal calibration process between the MMW radar and the monocular camera, and presents the construction of a monocular distance estimation model based on a target detection algorithm; Section 3 presents a dual-constrained matching algorithm for matching the targets detected by MMW radar and the monocular camera; Section 4 details the design and implementation of the AFEKF algorithm; Section 5 validates and analyzes the effectiveness of the proposed algorithm through a series of experiments.

## 2. Multi-Sensor Spatio-Temporal Calibration and Monocular Ranging Model Construction

This chapter aims to address the alignment issues between MMW radar and monocular cameras in terms of sampling frequency and spatial coordinate systems, and to construct a ranging model based on the YOLOv5 object detection algorithm, laying the foundation for the subsequent fusion of data from the two types of sensors.

### 2.1. Temporal Calibration

**Figure 1:**
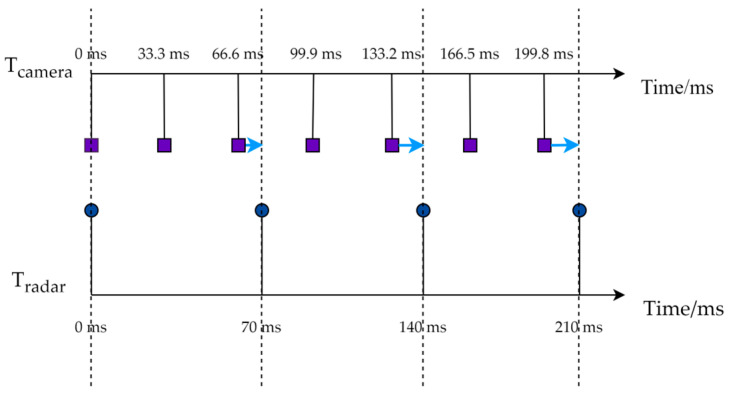
Temporal calibration of camera and radar.

To ensure the consistency of the detection data from the MMW radar and the monocular camera, synchronization of the sampling times is necessary. The sampling period of the MMW radar is 70 ms and the sampling frequency of the monocular camera is 30 fps, corresponding to a sampling period of approximately 33.3 ms. This paper assigns independent timestamps for the MMW radar and the monocular camera, denoted as $T_{radar}$ and $T_{camera}$, respectively. Using the radar’s sampling period as the reference frame, the timestamps of the camera are adjusted to be backward-compatible with the radars sampling time, as shown in Figure 1.

### 2.2. Spatial Calibration

The target data detected by the MMW radar and the monocular camera are located in different coordinate systems in the perception space. To achieve spatial matching of the targets, the target coordinate points detected by the radar are associated with the target pixel points in the camera image [33,34,35], thereby determining the spatial correspondence of the same target between the two sensors.

**Figure 2:**
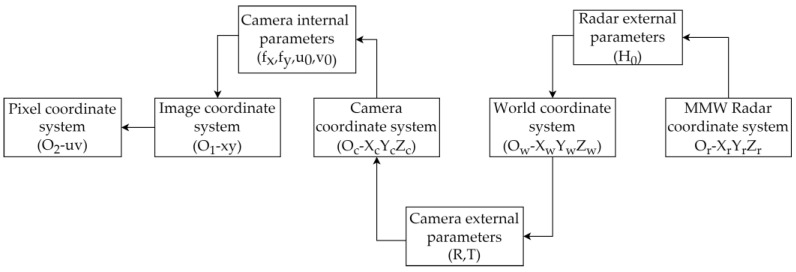
Translation relationships between different coordinate systems.

Figure 2 illustrates the spatial transformation relationship between the MMW radar coordinate system and the pixel coordinate system. The radar coordinate system maps its detected points to a unified world coordinate system using extrinsic parameters. The pixel coordinate system, based on both intrinsic parameters and extrinsic parameters, maps image plane pixels to the same world coordinate system. By establishing transformation relationships from both radar and camera coordinate systems to the world coordinate system, a direct mapping between the two sensor coordinate systems can be derived, enabling spatial alignment and coordinate unification across sensors.

**Figure 3:**
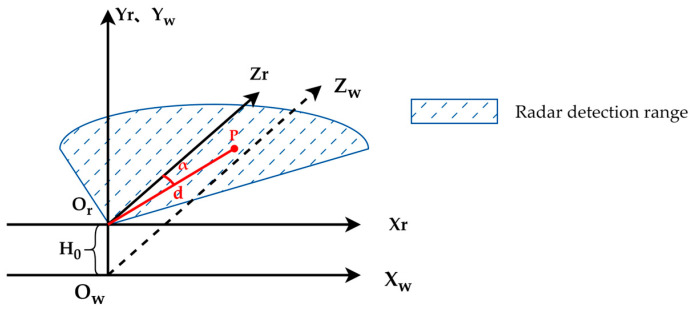
Relationship between MMW radar coordinate system and world coordinate system.

The relationship between the MMW radar coordinate system and the world coordinate system constructed in this paper is illustrated in Figure 3. The MMW radar coordinate system is denoted as $X_{r}Y_{r}Z_{r}-O_{r}$, while the world coordinate system is denoted as $X_{w}Y_{w}Z_{w}-O_{w}$, directly below it. The transformation relationship between them can be expressed as follows:

(1) $$\left\{\begin{array}{c} X_{w}=d\sin\alpha \\ Y_{w}=-H_{0}\\ Z_{w}=d\cos\alpha \end{array}\right.$$

The distance between the $X_{r}Y_{r}Z_{r}-O_{r}$ and the $X_{w}Y_{w}Z_{w}-O_{w}$ is $H_{0}$. The distance of the target point P is d, and the angle of yaw is α.

**Figure 4:**
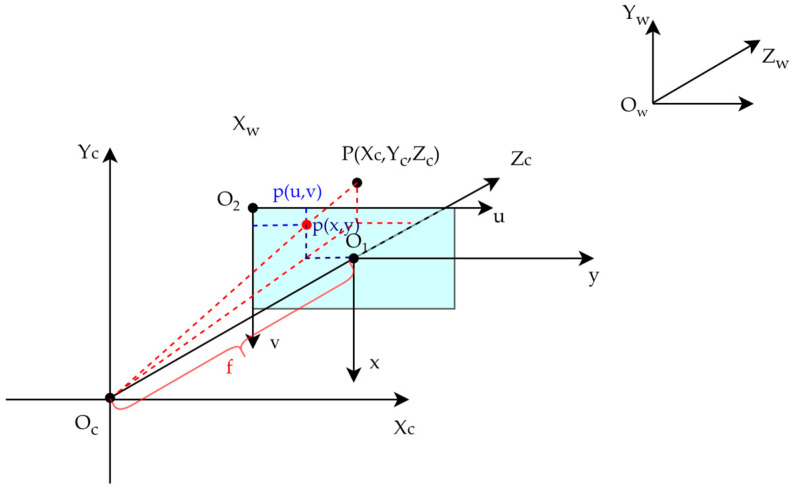
Relationship between pixel coordinate system, image coordinate system, and camera coordinate system.

The transformation between the world coordinate system and the camera coordinate system is described by the camera’s extrinsic parameters, representing a rigid-body transformation of the target’s position relative to the camera frame in three-dimensional space. The relationship between the pixel coordinate system and the camera coordinate system is illustrated in Figure 4. The mapping from the camera coordinate system to the image coordinate system follows the ideal pinhole camera model, in which three-dimensional points are projected onto the imaging plane through perspective projection. The transformation from the image coordinate system to the pixel coordinate system is defined by the camera’s intrinsic parameters. The formulae for the aforementioned coordinate transformations are presented in Table 1, and detailed derivations can be found in [36].

Based on the above process, the conversion relationship between the pixel coordinate system and the world coordinate system is shown as follows:

(2) $$Z_{c}\left[\begin{array}{c} u\\ v\\ 1 \end{array}\right]=\left[\begin{array}{llll} f_{x} & 0 & u_{0} & 0\\ 0 & f_{y} & v_{0} & 0\\ 0 & 0 & 1 & 0 \end{array}\right]\left[\begin{array}{cc} R & T\\ 0^{T} & 1 \end{array}\right]\left[\begin{array}{c} X_{w}\\ Y_{w}\\ Z_{w}\\ 1 \end{array}\right]=M_{1}M_{2}\left[\begin{array}{c} d\sin\alpha \\ -H_{0}\\ d\cos\alpha \\ 1 \end{array}\right]$$

where $Z_{c}$ represents the coordinate of the target along the Z-axis in the camera coordinate system, indicating the depth information of the target relative to the camera. $M_{1}$ and $M_{2}$ are the intrinsic and extrinsic matrices, respectively. $M_{1}$ includes information such as the focal length, principal point coordinates, and pixel scale, which describes the geometric properties of the camera’s imaging system. $M_{2}$ contains the rotation matrix and the translation vector, describing the position and orientation of the camera in world space.

**Figure 5:**
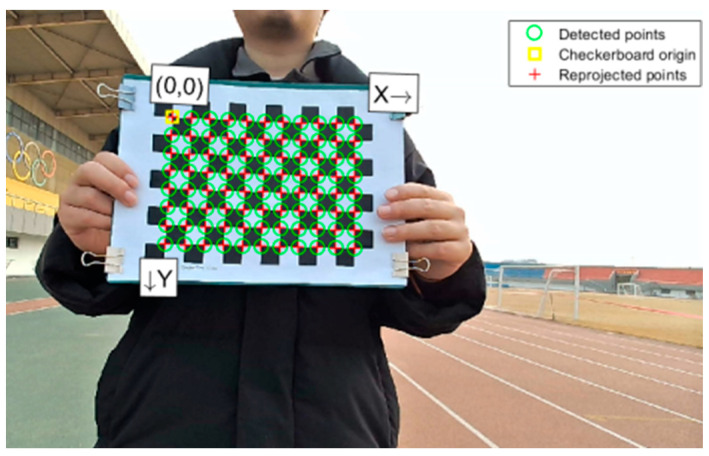
Checkerboard grid during calibration.

After introducing the coordinate transformation formulae, the camera’s intrinsic and extrinsic parameters are obtained through camera calibration. As shown in Figure 5, 20 images of a checkerboard taken from different angles were captured for camera calibration, with each checkerboard square measuring 15 mm.

After importing these images into the MATLAB camera calibration toolbox (Version: R2020b), the camera’s intrinsic parameter matrix $M_{1}$ was computed. The extrinsic parameter matrices $M_{2}$, which describe the relative pose and position of the camera for each image, varied across the different images. Based on the experimental calibration, specific images corresponding to particular poses were selected to compute the camera’s extrinsic parameters. The calibration results of the camera’s internal and external references are shown as follows:

(3) $$M_{1}=\left[\begin{array}{llll} 943.7591 & 0 & 964.7916 & 0\\ 0 & 943.7330 & 544.1173 & 0\\ 0 & 0 & 1 & 0 \end{array}\right]$$

(4) $$M_{2}=\left[\begin{array}{cccc} 0.9996 & -0.0193 & -0.0225 & 0\\ 0.0230 & 0.9835 & 0.1796 & -120\\ 0.0187 & -0.1800 & 0.9835 & 0\\ 0 & 0 & 0 & 1 \end{array}\right]$$

### 2.3. Monocular Ranging Model Based on Yolov5

Considering the computational resource requirements in practical applications, this paper adopts an efficient single-stage object detection algorithm. The YOLO [37] series of object detection algorithms have been widely applied in the field of computer vision due to their outstanding efficiency and real-time performance. This paper selects YOLOv5 as the object detection algorithm. Compared to previous generations of YOLO algorithms, YOLOv5 shows significant improvements in both detection speed and the performance of small object detection in complex environments [38,39,40]. In this section, a monocular distance estimation model is built based on the YOLOv5 object detection algorithm, incorporating geometric constraints and prior experimental conditions. This model provides data support for subsequent cross-modal matching and data fusion between the camera and radar.

**Figure 6:**
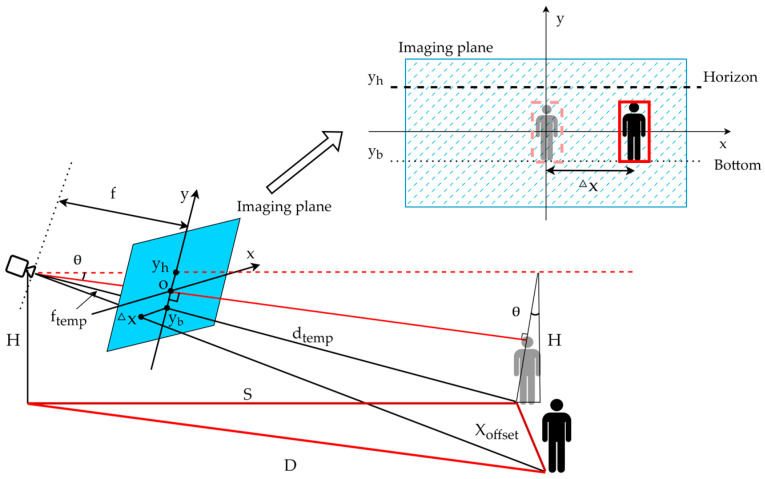
Monocular ranging model.

The detection boxes output by the object detection algorithm provide the pixel coordinates of the target in the image and based on this a monocular camera geometric distance measurement model is constructed, as shown in Figure 6. When the target is directly in front of the camera, as illustrated by the gray icon in the figure, the distance S is given by the following formulae:

(5) $$f=\frac{f_{x}+f_{y}}{2}$$

(6) $$\frac{f/\cos\theta }{S+H\cdot \tan\theta }=\frac{y_{h}-y_{b}}{H/\cos\theta }$$

(7) $$S=\frac{f\cdot H}{(y_{h}-y_{b})\cdot \cos^{2}\theta }-H\cdot \tan\theta$$

where the height of the camera is *H* and the angle with the horizontal plane is *θ*. Point o represents the camera’s optical center, $y_{h}$ is the coordinate of the horizon on the image plane’s *y*-axis, $y_{b}$ is the vertical coordinate of the bottom of the target detection box output by YOLOv5 on the image plane, and f is the focal length of the camera as measured by Equation (3).

When the target undergoes lateral displacement, as shown by the black icon in Figure 6, the lateral displacement of the target is denoted as $X_{offset}$. Using the midpoint of the bottom of the detection box as a reference, the lateral displacement on the image plane is denoted as △x. In this case, the distance *D* is given by the following formulae:

(8) $$\frac{△x}{X_{offset}}=\frac{f_{temp}}{d_{temp}}=\frac{y_{h}-y_{b}}{H/\cos\theta }$$

(9) $$X_{offset}=\frac{△x\cdot H}{(y_{h}-y_{b})\cdot \cos\theta }$$

(10) $$D=\sqrt{S^{2}+X_{offset}^{2}}$$

**Figure 7:**
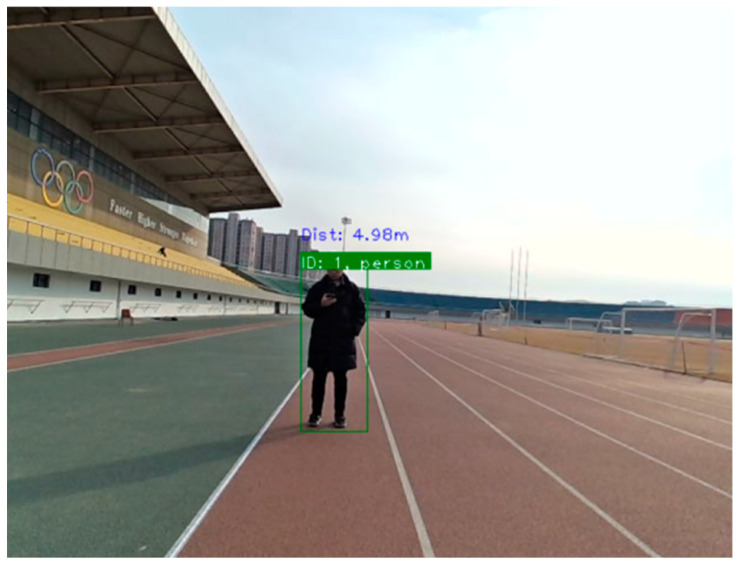
Preliminary validation of the monocular distance measurement model (model output: 4.98 m, actual: 5 m).

Figure 7 shows the preliminary validation of the monocular distance measurement model, illustrating the detection box output by the object detection algorithm of the monocular camera and its corresponding distance when the target is located 5 m away.

This chapter focused on the alignment of data between the MMW radar and the monocular camera, detailing the implementation of temporal synchronization, spatial calibration, and a monocular distance estimation model. Temporal synchronization is achieved by using the radar’s sampling frequency as a reference to align the asynchronous sensor data. Spatial alignment is accomplished through a coordinate transformation based on the camera’s intrinsic and extrinsic parameters, ensuring consistency in spatial observation. Additionally, a monocular distance estimation model was built, leveraging object detection bounding boxes and imaging geometry. These methodologies establish the foundation for subsequent target association between the radar and camera, as well as the fusion of multi-source ranging data.

## 3. Target Matching

Given the differences in detection mechanisms, field of view, and perception capabilities between MMW radar and monocular cameras, the detection results for the same scene may be inconsistent. To achieve effective fusion of multi-sensor information it is necessary to solve the problem of matching target detection results, that is, to ensure that the detection data of the same target under different sensors can be accurately associated [41]. To this end, this study employs a dual-constraint matching algorithm based on intersection over union (IOU) and Euclidean distance to address the data association problem between MMW radar and monocular camera. By jointly considering the overlap in the image space and the distance consistency in the three-dimensional space, the algorithm achieves accurate cross-modal sensor data matching.

### 3.1. Determination of Radar Projection Area

The MMW radar maps the two-dimensional position of a detected target from the radar coordinate system onto the image plane through a spatial coordinate transformation, as described in Equation (2). Assuming the projected position of the target on the image plane is denoted by $\left(u_{r},v_{r}\right)$, a corresponding region of interest (ROI) is generated by centering at this coordinate and expanding it according to the target’s physical dimensions in the real-world scene. This approach ensures that the ROI fully encompasses the spatial extent of the target. The calculation formulae are as follows:

(11) $$\left\{\begin{array}{cc} w_{r}=\frac{W_{p}}{Z_{r}}\cdot f_{x}, & h_{r}=\frac{H_{p}}{Z_{r}}\cdot f_{y}\\ u_{b}=u_{r}-\frac{w}{2}, & v_{b}=v_{r}-\frac{h}{2} \end{array}\right.$$

In this paper, pedestrians are selected as the detection target, with the assumption that the height of the pedestrian $H_{p}$ is 1.75 m and the width $W_{p}$ is 0.5 m. In the image coordinate system, $f_{x}$ and $f_{y}$ represent the focal lengths in the x and y directions, respectively. $Z_{r}$ is the distance of the target in the longitudinal direction of the MMW radar, and w and h are the width and height of the generated ROI, respectively. $\left(u_{b},v_{b}\right)$ are the pixel coordinates of the top-left corner of the ROI.

### 3.2. Dual-Constraint Target Matching Algorithm

After projecting the radar data onto the image plane, a dual-constraint matching algorithm is employed to identify valid target pairs between the radar and camera. Since MMW radar measurements typically contain a large number of irrelevant points and exhibit sparse spatial distribution, whilst camera-based object detection yields targets with higher confidence scores, a unidirectional matching strategy is adopted in this study. Specifically, the target set detected by the camera is fixed as the reference, and each radar-detected target is sequentially evaluated against the camera targets using the defined matching constraints.

**Figure 8:**
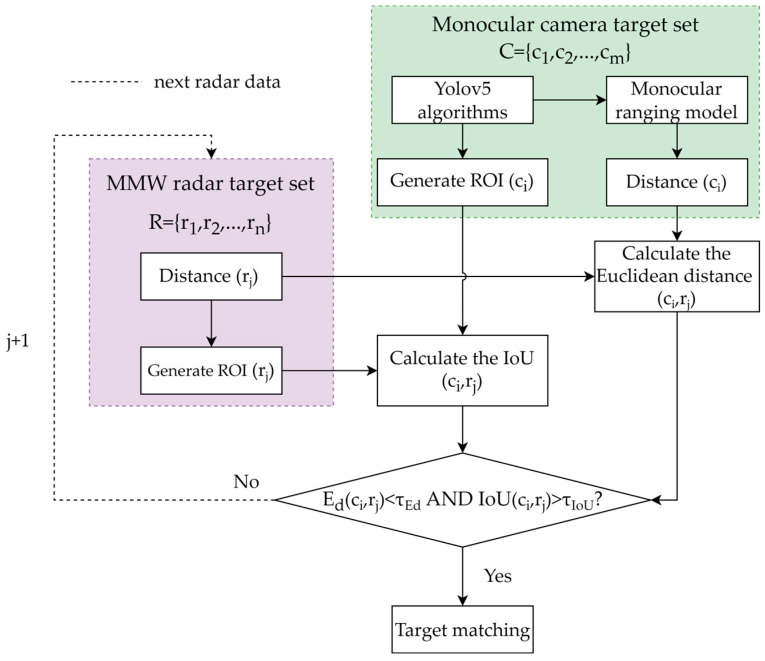
Pixel-distance joint dual-constraint matching algorithm.

As shown in Figure 8, each sensor stores its detection results in a corresponding set for each frame. Each target in the set is characterized by two key attributes: distance and ROI. For radar targets, the distance is directly measured by the radar, and the ROI is computed using Equation (11). For camera targets, the distance is estimated using Equations (5) to (10) outlined in Section 2.3., while the ROI is determined using the YOLOv5 object detection algorithm. The target matching procedure is as follows:

Step 1: Input the target sets obtained from the MMW radar and the monocular camera.

Step 2: For each radar target, compute the Euclidean distance and IOU between it and all camera targets, which then serve as the constraints for matching. The calculation formulae are as follows:

(12) $$E_{d}(c_{i},r_{j})=\left|d_{c,i}-d_{r,j}\right|$$

(13) $$IoU(c_{i},r_{j})=\frac{S_{r,i}\cap S_{c,j}}{S_{r,i}\cup S_{c,j}}$$

where $E_{d}(c_{i},r_{j})$ denotes the Euclidean distance between the *i*-th camera $c_{i}$ target and the *j*-th radar target $r_{i}$. $S_{r}$ represents the ROI of the radar target projected onto the image plane through spatial coordinate transformation, while $S_{c}$ denotes the bounding box obtained by the object detection algorithm from the camera. A camera target and a radar target are considered successfully matched if they simultaneously satisfy the constraints $IoU(c_{i},r_{j})>\tau_{IoU}\;\mathrm{and}\;E_{d}(c_{i},r_{j})<\tau_{Ed}$. The parameters $\tau_{IoU}$ and $\tau_{Ed}$ are the predefined thresholds for the IOU and Euclidean distance, respectively.

Step 3: Output the final matching result.

**Figure 9:**
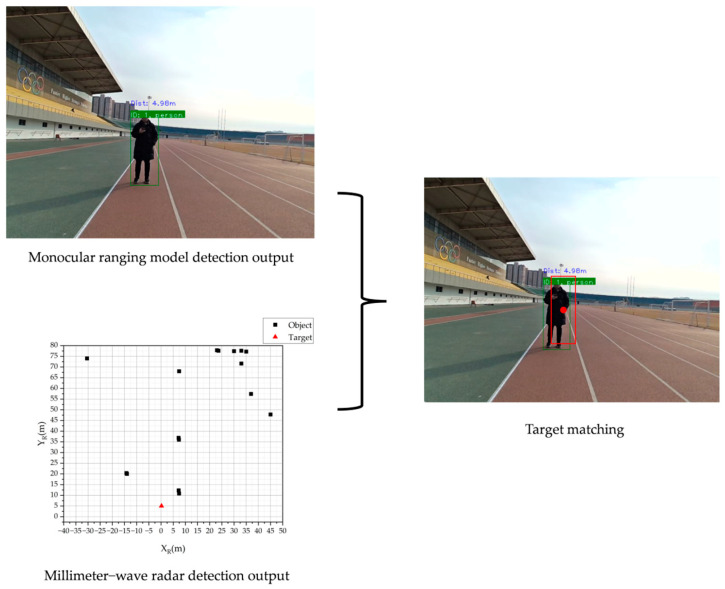
Schematic of the successful matching of the MMW radar and monocular camera.

Considering the measurement errors inherent in both sensors, this paper sets the threshold for the Euclidean distance $E_{d}$ to 1 m and the threshold for the IOU to 0.5. A match is considered successful when the results from both the MMW radar and the camera simultaneously meet these thresholds. Figure 9 shows a scenario where the radar and the camera successfully match.

## 4. Improved Data Fusion Algorithm

When the MMW radar and the camera successfully match, this paper employs an improved extended Kalman filter (EKF) algorithm to fuse the measurement data from both sensors, aiming to enhance the system’s distance measurement accuracy. Building upon the traditional extended Kalman filter, this paper introduces the following two key improvements:

1. Dynamic error functions are proposed to correct the system’s error covariance matrix, thereby more accurately reflecting the real-time variations in the errors;
2. Adaptive weight allocation formulae are proposed to add weight to the measurement inputs and Kalman gain in the update step, thereby optimizing the information weight distribution during the fusion process

### 4.1. Motion Model Construction

In this paper, pedestrians with uniform velocity motion (UVM) are used as the detection target. It is assumed that the target’s state vector consists of three parts: *d* representing the target’s distance, *v* representing the target’s velocity, and *α* representing the target’s yaw angle, as shown in the following:

(14) $$x=\left[d,v,\alpha \right]$$

In the uniform velocity motion model, the initial position of the target is set as $\left[d_{0},v_{0},\alpha_{0}\right]$, and the time step is *T*. The changes in the components of the state vector are as follows:

(15) $$\left\{\begin{array}{c} d_{k+1}=\sqrt{{\left(d_{0}\sin\alpha_{0}\right)}^{2}+{\left(v_{k}T+d_{0}\cos\alpha_{0}\right)}^{2}}\\ v_{k+1}=v_{k}\\ \alpha_{k+1}=\arctan\frac{d_{0}\sin\alpha_{0}}{v_{k}T+d_{0}\cos\alpha_{0}} \end{array}\right.$$

where *k* refers to the current moment and *k* + 1 to the next moment.

By calculating the Jacobian matrix, the system’s motion state equation is linearized to obtain the state transition matrix $A_{k}$, as shown in the following:

(16) $$A_{k}=J_{F}(d,v,\alpha )$$

### 4.2. Measurement Error Functions

As the sensor measurement errors fluctuate differently with the target’s position, using a fixed error value for compensation will result in instability in the filtering results. To address this, this paper analyzes the measurement errors of the sensor over a period of time and fits a function relating the errors to the target distance, enabling dynamic correction of the measurement errors. Specifically, through experimental data fitting, this paper proposes two mathematical functions that describe the relationship between the measurement errors of the monocular camera and the MMW radar and the target’s distance.

The distance measurement error of the monocular camera increases non-linearly with the target’s distance. At close distances, the error is small and changes smoothly, while at longer distances, due to limitations in pixel accuracy and insufficient image resolution, the error increases progressively. This growth pattern of the error follows the characteristics of exponential growth. Therefore, this paper uses a power function to fit the relationship between the monocular camera’s measurement error and the target’s distance, as shown in the following:

(17) $$e_{camera}(d)=a\cdot d^{b}+c$$

where *a*, *b*, and *c* are the parameters to be fitted, *d* represents the distance, and $e_{camera}(d)$ denotes the camera’s distance measurement error.

The distance measurement error of the MMW radar is influenced by multiple factors, including signal reflection, noise, the target’s surface characteristics, and environmental interference. At close distances, the radar error is small and changes smoothly. However, as the target distance increases, the error gradually increases, and due to the complex factors involved in the radar ranging process the error exhibits a more complex non-linear variation. The trend of error growth in different distance intervals is not a simple linear or exponential relationship but rather shows a progressively accumulating characteristic as the distance changes. Therefore, a polynomial error function is adopted, as shown in the following:

(18) $$e_{radar}(d)=a\cdot d^{2}+b\cdot d+c$$

where *a*, *b*, and *c* are the parameters to be fitted, *d* represents the target’s distance, and $e_{radar}(d)$ denotes the distance measurement error of MMW radar.

### 4.3. Adaptive Weighting Allocation Formulae

Sensor fusion systems typically involve multiple inputs. Therefore, in this study, the adaptive fuzzy weighting allocation formulae are proposed to adjust the information weight of different sensors, thereby enhancing the overall perception accuracy and robustness of the system. Specifically, these formulae monitor the error conditions of the camera and radar in real-time and flexibly allocate sensor weights based on the variations in their measurement accuracy.

Defining the confidence level $C_{i}(k)$ in sensor measurement is done by using Gaussian functions [42], as shown in the following:

(19) $$C_{i}(k)=\frac{1}{\sqrt{2\pi }e_{i}(z_{pred}(k))}\exp(-\frac{{\left(z_{i}(k)-z_{pred}(k)\right)}^{2}}{2e_{i}^{2}(z_{pred}(k))})$$

where *i* denotes the sensor type, $z_{i}(k)$ is the actual measurement value of the sensor, $z_{pred}(k)$ is the predicted value based on the state transition matrix, and $e_{i}(z_{pred}(k))$ represents the measurement error of sensor *i* at its current location. The confidence level set $\left\{C_{i}(k)\right\}$ is normalized to construct the fuzzy membership function $\mu_{i}(k)$. The specific calculation formula is given as follows:

(20) $$\mu_{i}(k)=\frac{C_{i}(k)}{\sum_{i=1}^{n}C_{i}(k)}$$

To accommodate the changes in sensors during the measurement process, an adjustment factor $\lambda_{i}(k)$ is designed based on the direct deviation between the sensor measurement and the predicted value to quantify the degree of deviation of the sensor measurement value that enables dynamic adjustment of the weights, as shown in the following:

(21) $$\lambda_{i}(k)=\frac{1}{(\varepsilon +\left|z_{i}(k)-z_{pred}(k)\right|)\cdot \sum_{j=1}^{n}\frac{1}{\varepsilon +\left|z_{j}(k)-z_{pred}(k)\right|}}$$

where the small positive value ε is introduced to avoid division by zero.

By combining the fuzzy membership function and the dynamic adjustment factor, the final weight is obtained through normalization. The calculation formula is given as follows:

(22) $$w_{i}(k)=\frac{\mu_{i}(k)\cdot \lambda_{i}(k)}{\sum_{i=1}^{n}\mu_{i}(k)\cdot \lambda_{i}(k)}$$

The weighted weights $w_{radar}$ and $w_{camera}$ of the MMW radar and the monocular camera are obtained through the adaptive fuzzy weighting formulae. Based on these weights, the sensor measurement values and the Kalman gain are weighted and the result is used as the observation input for the extended Kalman filter.

### 4.4. Improved Extended Kalman Filter

The extended Kalman filter (EKF) relies on known mathematical models and is widely applied in the estimation and state prediction of dynamic systems [43] as it continuously optimizes the state estimate through prediction and update steps. In this paper, the dynamic error functions and the adaptive fuzzy weighting allocation formulae are incorporated into the extended Kalman filter to enhance the accuracy of multi-sensor data fusion.

Equation (16) constructs the system’s state transition matrix $A_{k}$, and the system’s state prediction $x_{k|k-1}^{-1}$ is shown as follows:

(23) $$x_{k|k-1}^{-}=A_{k}x_{k-1|k-1}+B_{k}u_{k-1}$$

where $x_{k-1|k-1}$ represents the optimal estimate of the system at the previous time step, $B_{k}$ is the control input matrix, and $u_{k}$ is the control input vector.

P is the covariance matrix of the system state estimation error, which represents the uncertainty of the system state prediction [44], as shown in the following:

(24) $$P_{k|k-1}^{-}=A_{k}P_{k-1|k-1}A_{k}^{T}+Q$$

where $P_{k-1|k-1}$ is the updated state estimation error covariance matrix of the system at the last moment of time and Q is the process noise covariance matrix which describes the randomness or uncertainty of the system model itself.

In this algorithm, the measurement error is dynamically updated through the dynamic error functions, where at time step k the predicted value from the motion equation is used as the input and the error of sensor *i* is represented as $R_{i,k}$, as shown in the following:

(25) $$R_{i,k}=e_{i,k}^{2}(x_{k|k-1}^{-})$$

The Kalman gain determines the weighted ratio between the predicted value and the measurement value. It optimizes the combination of the prediction and measurement results by balancing the $R_{i,k}$ and $P_{k|k-1}^{-}$. The calculation formula is as follows:

(26) $$K_{i,k}=P_{k|k-1}^{-}H_{k}^{T}{\left(H_{k}P_{k|k-1}^{-}H_{k}^{T}+R_{i,k}\right)}^{-1}$$

where $K_{i,k}$ donates the Kalman gain of the *i*-th sensor at time step *k* and *H* is the observation matrix which maps the system’s state vector to the measurement space. Since this experiment only observes distance, the observation matrix H is given by the following:

(27) $$H_{k}=\left[\begin{array}{ccc} 1 & 0 & 0 \end{array}\right]$$

For the Kalman gain $K_{i,k}$ of different sensors and the measurement value $z_{i,k}$, the $w_{i,k}$ is calculated based on Equations (20)–(22), as shown in the following:

(28) $$K_{k}=\sum_{i=1}^{n}w_{i,k}\cdot K_{i,k}$$

(29) $$z_{k}=\sum_{i=1}^{n}w_{i,k}\cdot z_{i,k}$$

The optimal state estimate of system at the current time step, based on the predicted value and the measurement value, is $x_{k|k}$ and is calculated using the following formula:

(30) $$x_{k|k}=x_{k|k-1}^{-}+K_{k}(z_{k}-H_{k}x_{k|k-1}^{-})$$

Finally, the system state estimation error covariance matrix is updated to lay the groundwork for calculating the system’s predicted state estimation error covariance at the next time step, which is calculated as follows:

(31) $$P_{k|k}=(I-K_{k}H_{k})P_{k|k-1}^{-}$$

where *I* is the unit matrix.

### 4.5. Date Fusion Algorithm Process

The overall process of the AFEKF algorithm is outlined in Algorithm 1. The algorithm takes as its initial inputs the target’s motion model, the initial state, the observation matrix, the initial state estimation error covariance matrix, and the process noise covariance matrix. A two-level iterative loop structure is employed, and the outer loop traverses all time steps of the target’s motion and the inner loop sequentially performs the fuzzy processing and weight updating for each sensor’s data, ultimately achieving fusion-based estimation. The detailed steps are as follows:

Step 1: Define the measurement error functions for each sensor to provide the basis for subsequent processing.

Step 2: In the first inner loop, calculate the confidence level of each sensor at the time step k based on Equation (19), and generate the confidence set.

Step 3: In the second inner loop, the confidence levels of each sensor are normalized according to Equation (20) to obtain the fuzzy membership degrees. Subsequently, the corresponding correction factors are calculated using Equation (21) and the set $\left\{\mu_{i}(k),\lambda_{i}(k)\right\}$ containing the fuzzy membership degrees and correction factors for all sensors at the current time step is output.

Step 4: In the third inner loop, based on the fuzzy membership degrees and correction factors obtained in Step 3, the dynamic weight set $\left\{w_{i}(k)\right\}$ for all sensors at the current time step is calculated according to Equation (22).

Step 5: Output the predicted state $x_{k|k-1}^{-}$ and the predicted error covariance $P_{k|k-1}^{-}$ of the target at the current time step according to Equations (23) and (24).

Step 6: In the fourth inner loop, using matrices $x_{k|k-1}^{-}$ and $P_{k|k-1}^{-}$ from Step 5 as inputs, perform the following operations:

1. Calculate the measurement error $R_{i,k}$ of each sensor based on Equation (25).
2. Compute the local Kalman gain $K_{i,k}$ of each sensor based on Equation (26).
3. Using the weights obtained in Step 4, perform weighted fusion of all Kalman gains and measurements according to Equations (28) and (29) to obtain the global Kalman gain A and the global measurement Z.

Step 7: Substitute the weighted Kalman gain and measurement into Equation (30) to obtain the fusion result $x_{k|k}$ at time step k.

Repeat Steps 2–7 until all N observations of the target over the entire time period are processed. Finally, output the fusion result sequence $\left\{x_{k|k}\right\}$. (The explanation of the symbols in the above algorithm can be found in Table A1 of Appendix A).
**Algorithm 1.** Adaptive Fuzzy Extended Kalman Filter1.**Inputs:** $A_{k}$$,\;x_{0}$$,\;H,\;P_{0}$, Q**2.** **Initialize error functions:**$\begin{array}{l} e_{camera}(d)=a\cdot d^{b}+c\\ e_{radar}(d)=a\cdot d^{2}+b\cdot d+c \end{array}$3.**for** k = 1 to N **do**4.**---------------------------------------------------------------------------------------------------------------**5.**for** i = 1 to n **do**6.$C_{i}(k)=\frac{1}{\sqrt{2\pi }e_{i}(z_{pred}(k))}\exp(-\frac{{\left(z_{i}(k)-z_{pred}(k)\right)}^{2}}{2e_{i}^{2}(z_{pred}(k))})$7.i = i + 1**8.** **end for**9.**return** $\left\{C_{i}(k)\right\}$10.**---------------------------------------------------------------------------------------------------------------**11.**for** i = 1 to n **do**12.$\mu_{i}(k)=\frac{C_{i}(k)}{\sum_{i=1}^{n}C_{i}(k)}$13.Calculating dynamic factors and adjusting sensor weights:14.$\lambda_{i}(k)=\frac{1}{(\varepsilon +\left|z_{i}(k)-z_{pred}(k)\right|)\cdot \sum_{j=1}^{n}\frac{1}{\varepsilon +\left|z_{j}(k)-z_{pred}(k)\right|}}$15.i = i + 116.**end for**17.**return** $\left\{\mu_{i}(k),\lambda_{i}(k)\right\}$18.**---------------------------------------------------------------------------------------------------------------**19.**for** i = 1 to n **do**20.$w_{i}(k)=\frac{\mu_{i}(k)\cdot \lambda_{i}(k)}{\sum_{i=1}^{n}\mu_{i}(k)\cdot \lambda_{i}(k)}$21.i = i + 1**22.** **end for**23.**return** $\left\{w_{i}(k)\right\}$24.**---------------------------------------------------------------------------------------------------------------**25.Prediction:26.$x_{k|k-1}^{-}=A_{k}x_{k-1|k-1}+B_{k}u_{k-1}$27.$P_{k|k-1}^{-}=A_{k}P_{k-1|k-1}A_{k}^{T}+Q$28.Update:29.**---------------------------------------------------------------------------------------------------------------**30.**for** i = 1 to n **do**31.$R_{i,k}=e_{i,k}^{2}(x_{k|k-1}^{-})$32.$K_{i,k}=P_{k|k-1}^{-}H_{k}^{T}{\left(H_{k}P_{k|k-1}^{-}H_{k}^{T}+R_{i,k}\right)}^{-1}$33.$K_{k}=\sum_{i=1}^{n}w_{i,k}\cdot K_{i,k}$, $z_{k}=\sum_{i=1}^{n}w_{i,k}\cdot z_{i,k}$34.i = i + 135.**end for**36.**return** $K_{k},z_{k}$37.**---------------------------------------------------------------------------------------------------------------**38.$x_{k|k}=x_{k|k-1}^{-}+K_{k}(z_{k}-H_{k}x_{k|k-1}^{-})$39.$P_{k|k}=(I-K_{k}H_{k})P_{k|k-1}^{-}$40.k = k + 141.**end for**42.**Output:** $\left\{x_{k|k}\right\}$

## 5. Experiment

### 5.1. Experiment Hypotheses

In this section, a uniformly moving target is simultaneously measured by both the camera and radar. Based on multiple sets of experimental data, the performance of the proposed AFEKF algorithm is evaluated and compared with the EKF and IVW algorithms to assess its advantages in terms of ranging accuracy and robustness. To systematically validate the effectiveness of the proposed algorithm, the following experimental hypotheses are established:

1. AFEKF can adaptively adjust the weight allocation based on variations in sensor measurements through an adaptive fuzzy weighting mechanism, thereby enhancing the adaptability of the fusion process to changes in sensor data.
2. In the task of fusing ranging data for dynamic targets, the AFEKF algorithm achieves higher fusion accuracy compared to the EKF and IVW algorithms.

### 5.2. Experimental Environment and Equipment

**Figure 10:**
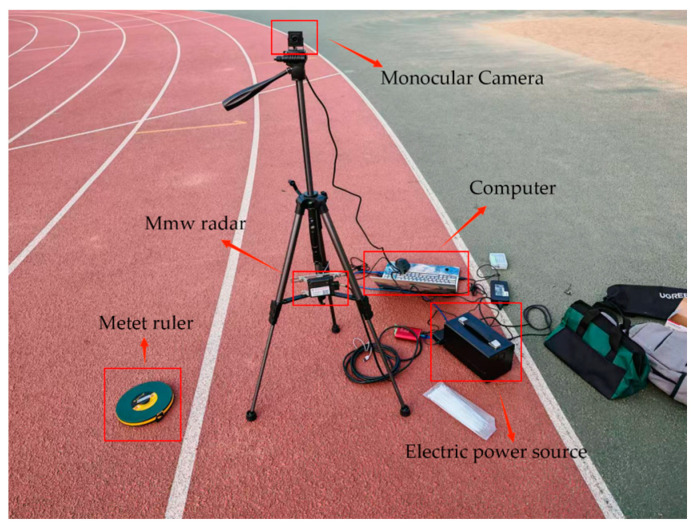
Equipment installation schematic.

The experimental setup is illustrated in Figure 10. A Continental ARS408-21 MMW radar operating at a transmission frequency of 76–77 GHz is employed, along with an SY011HD-V1 industrial-grade high-definition camera featuring a resolution of 1920 × 1080 pixels and a frame rate of 60 FPS. The two sensors transmit data to the computer via a CAN bus and a USB interface, respectively. During the experiment, a high-precision engineering measuring tape is used to calibrate the true position of the target, thereby establishing the sensor ranging error functions. To minimize system calibration errors during device installation, the camera is rigidly mounted directly above the MMW radar, simplifying the modeling of the spatial relationship between the two sensors. Furthermore, to maintain consistency with the coordinate system definitions provided in Section 2., the radar is vertically installed on the ground, with the mounting point at the bottom of the radar defined as the origin of the world coordinate system. The MMW radar is installed at a height of 0.34 m, and the monocular camera is at 1.2 m.

### 5.3. Dynamic Error Functions Construction

In this paper, multiple sampling points are set within the range of 5 to 50 m, with an interval of 5 m. At each sampling point, 20 frames of data are collected, and the average value is taken as the final measurement for that point. At each sampling point, the error between the final measurement value and the true value is calculated, and curve fitting analysis is performed on the error data. Table 2 shows the measurement values and true values of the MMW radar and the monocular camera at different sampling points.

**Figure 11:**
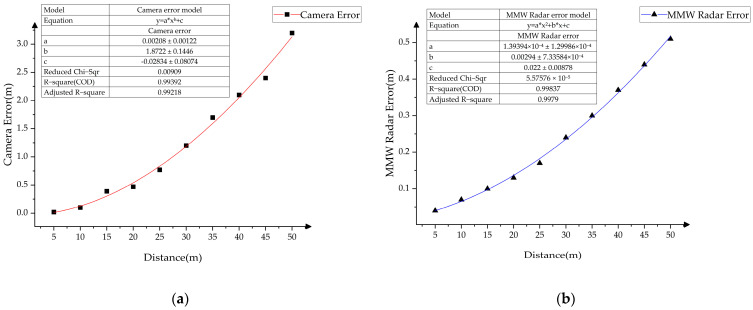
Sensor measurement error functions. (**a**) Camera measurement error function; (**b**) MMW radar measurement error function.

This experiment integrates the measured data into the error functions defined in Section 4.2. for parameter fitting, thereby constructing functions that characterize the variations in ranging errors for both the monocular vision system and the MMW radar as a function of target distance. These error functions enable real-time quantification of measurement uncertainties for moving targets at varying ranges, facilitating a dynamic adjustment mechanism for the measurement error covariance matrix based on the target’s position dynamics. The results are presented in Figure 11a,b.

### 5.4. Improved Extended Kalman Filter Verification

To validate the effectiveness of the AFEKF proposed in this paper, we compare it with the inverse variance weighting (*IVW*) and traditional extended Kalman filter (EKF) algorithms.

The core idea of *IVW* is to adjust the weight of each sensor’s measurement based on its observation accuracy, as shown in the following:

(32) $$w_{i}(IVW)=\frac{1/\sigma_{i}^{2}}{\sum_{i=1}^{n}1/\sigma_{i}^{2}}$$

where $\sigma_{i}^{2}$ represents the measurement variance of the *i*-th sensor and $w_{i}$ denotes the weight of the *i*-th sensor calculated by *IVW*.

By taking the inverse of each sensor’s measurement error and normalizing it the weight allocation for each sensor is determined, ensuring that sensors with higher measurement accuracy contribute more to the final result in the calculation process. The traditional extended Kalman filter constructs the measurement error covariance matrix using fixed error values. The covariance matrix of each sensor’s measurement error reflects the uncertainty of that sensor’s measurement, and the filter adjusts the influence of each sensor in the fusion process based on this information. In this paper, the average measurement error values of the MMW radar and the monocular camera at different distances are used as fixed error values, as shown in Table 3.

This paper uses high-precision GPS data as the true positions, and the experiment is conducted in an open playground on the campus. The experimental subject starts from a distance of 5 m and walks at a constant speed in a straight line along the track to a distance of 50 m. The MMW radar and the monocular camera record the changes in distance, respectively. To ensure the reliability of the experiment results, 15 sets of data were tested and the root mean square error (*RMSE*) between the fusion values from the three algorithms and the true values at each timestamp was calculated as the evaluation metric. The *RMSE* is calculated as follows:

(33) $$RMSE=\sqrt{\frac{1}{n}\sum_{i=1}^{n}{\left(x_{i}-{\hat{x}}_{i}\right)}^{2}}$$

where *n* is the number of the data and $x_{i}$ and ${\hat{x}}_{i}$ represent the *i*-th measured value and the true value, respectively.

**Figure 12:**
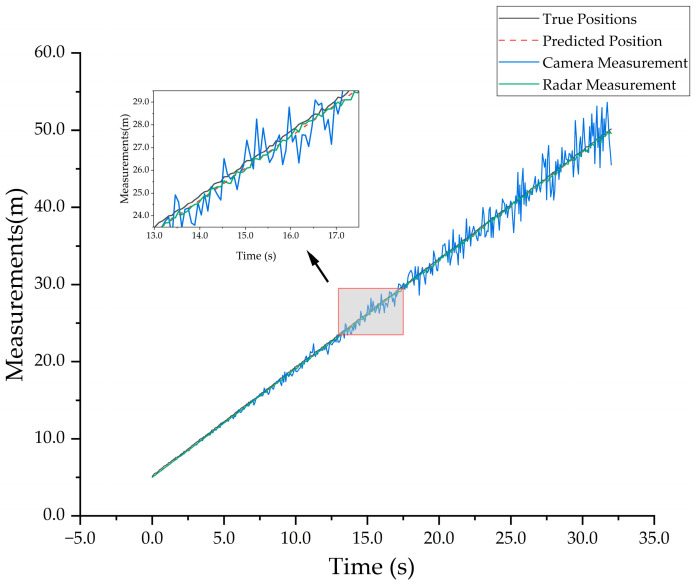
Measurement of sensors and true values.

Figure 12 shows the comparison between the measured values and the true values in the experiment. During the measurement process, the target’s motion caused fluctuations in the detection boxes output by the target detection algorithm, leading to instability in the monocular distance measurement results.

**Figure 13:**
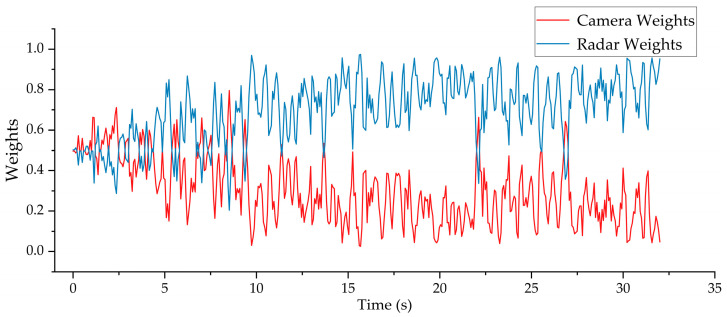
Changes in the weights of camera and radar during fusion in a dataset.

Figure 13 illustrates the variation in sensor weights during the ranging data fusion process for a representative set of experimental data. It can be observed that the weights of the radar and camera continuously fluctuate throughout the fusion process, reflecting the adaptive response of the fusion algorithm to the changes in the residuals between the measurement and prediction results. The algorithm adaptively adjusts the sensor weights based on the magnitude of the residuals between each measurement and the predicted state. When the residual between a sensor’s observation and the system’s predicted value is large, indicating higher uncertainty in the sensor’s current measurement, its weight is accordingly reduced to mitigate its negative impact on the fusion result. Conversely, when the residual is small, suggesting a reliable measurement, the sensor’s weight is appropriately increased to enhance its contribution to the fused output.

**Figure 14:**
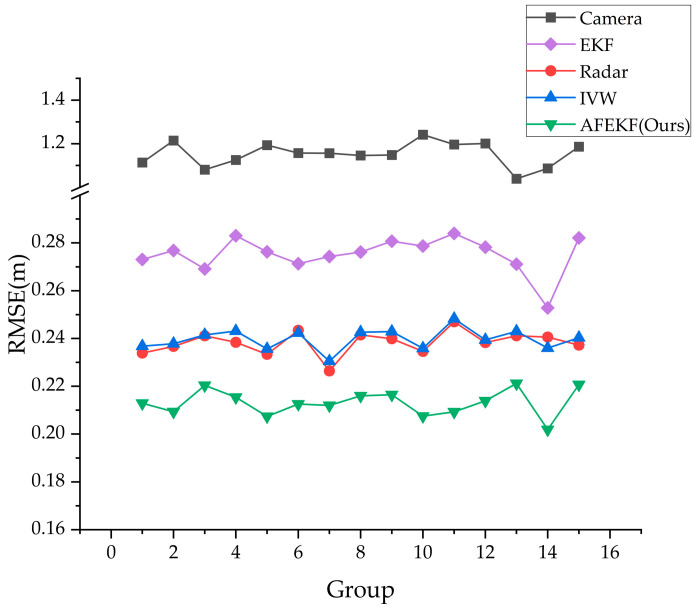
Comparison of RMSE across different algorithms for 15 datasets.

Figure 14 shows the comparison results of the three algorithms with the measurement errors from the MMW radar and the monocular camera for 15 sets of experimental data. All three algorithms effectively reduce the monocular camera distance measurement error. However, compared with the other two algorithms, EKF exhibits relatively weaker performance in error suppression, primarily due to the inherent measurement inaccuracies of monocular vision-based ranging systems. As a result, the accuracy of the fused data does not significantly surpass that of the original radar measurements. Although the IVW algorithm effectively reduces the ranging errors of the visual system to some extent, its optimization capability is still constrained by sensor characteristics. This algorithm can only converge the fusion errors to a level close to the radar measurement errors and does not achieve further improvements in accuracy. In contrast, AFEKF not only significantly mitigates the ranging errors of the camera system but also optimizes the fused errors to a level below the ranging errors of the radar system. Further analysis shows that the output of the IVW is more consistent with the trend of the MMW radar measurement values, indicating that this algorithm is more influenced by the sensor measurement values. The proposed AFEKF algorithm, however, shows more stable results across the 15 sets of measurement data, exhibiting higher robustness.

Table 4 lists the mean RMSE values for the 15 sets of measurement data. The results show that the mean RMSE of AFEKF is 0.2131, which is a 10.54% improvement over the raw radar measurement data, a 11.10% improvement over IVW, and a 22.57% improvement over EKF.

This experiment aims to measure the distance of a target in uniform linear motion and is designed based on the fusion of a monocular camera and MMW radar. A comparative analysis of fusion performance was conducted using the EKF, IVW, and the proposed AFEKF. Compared to IVW, AFEKF dynamically adjusts the weights of each sensor based on the measurement and prediction residuals, effectively suppressing the impact of single sensor errors on the fusion results. In contrast to the traditional EKF, AFEKF dynamically updates the measurement noise covariance according to changes in the target’s position, effectively compensating for the accuracy loss caused by the fixed error in practical applications.

Experimental results show that in multi-source heterogeneous perception systems, dynamically adjusting sensor measurement errors based on the motion state changes in moving targets and adaptively correcting weight distribution according to the fluctuations of sensor measurement values is an effective strategy to improve fusion accuracy. This study provides a new approach for multi-sensor data fusion in dynamic environments and shows strong applicability and potential in high-precision application scenarios such as autonomous driving and security surveillance.

## 6. Conclusions

This paper proposes a distance measurement method based on the fusion of the monocular camera and MMW radar. On the basis of spatio-temporal calibration, a monocular ranging model was constructed using the YOLOv5 object detection algorithm, enabling the camera to independently measure target distances. A dual-constraint matching strategy based on Euclidean distance and intersection over union (IoU) was adopted to achieve effective cross-modal matching between the monocular camera and radar. Furthermore, an adaptive fuzzy extended Kalman filter (AFEKF) algorithm was developed to fuse the distance measurements obtained from the two sensors.

Experiments were conducted on a common dataset, using the root mean square error (RMSE) between measurements and the ground truth as the evaluation metric. The comparison between the two sensors individually shows that the MMW radar achieved a lower RMSE, likely due to the radar’s direct electromagnetic wave-based measurement principle, whereas the camera’s performance is constrained by the precision of the bounding boxes generated by the detection algorithm. The proposed AFEKF algorithm, after fusing the measurements, achieved a lower RMSE compared to using either sensor alone. Moreover, comparisons with two traditional fusion algorithms, namely the extended Kalman filter (EKF) and inverse variance weighted (IVW) fusion, were conducted. The results demonstrate that while EKF and IVW did not significantly improve the fusion performance, AFEKF achieved the best results, with an average RMSE improvement of 11.10% and 22.57% compared to IVW and EKF, respectively. This improvement is attributed to AFEKF’s ability to dynamically adjust the weights of each sensor based on variations in measurement residuals, as well as its capability to adaptively update the measurement error covariance according to changes in the target’s position. These results validate the effectiveness of the proposed algorithm in dynamic environments and lay the foundation for future applications of multi-source perception data fusion in complex scenarios.

This study primarily focuses on distance fusion for targets undergoing uniform linear motion, with a reliance on the accuracy of the motion prediction model. However, in real-world applications target motion is often complex, making accurate prediction more challenging. Future work will focus on further optimizing the system’s motion modeling, exploring the use of deep learning or vehicular network technologies to achieve more accurate state predictions and verifying the proposed algorithm’s applicability and effectiveness under complex motion scenarios. Additionally, integrating more types of sensors to enable richer state perception of the targets will be another key research direction, aiming to enhance the system’s multidimensional sensing capabilities and overall accuracy.

## Figures and Tables

**Table 1:** The conversion formulae from world coordinates to pixel coordinates.

Step	Mathematical Formulation
World → Camera Coordinates	$P_{c}=R\cdot P_{w}+T$
Camera → Image Coordinates	$x=\frac{fX_{c}}{Z_{c}},y=\frac{fY_{c}}{Z_{c}}$
Image → Pixel Coordinates	$u=\frac{x}{dx}+u_{0},v=\frac{y}{dy}+v_{0}$

$P_{c}(X_{c},Y_{c},Z_{c})$ and $P_{w}(X_{w},Y_{w},Z_{w})$ represent the coordinates of the target point *P* in the camera coordinate system and the world coordinate system, respectively. *R* and *T* denote the rotation matrix and translation vector, respectively. (*x*,*y*) are the coordinates of the projection of *P* in the image coordinate system, and f denotes the camera focal length. (*u*,*v*) represent the corresponding pixel coordinates, while $\left(u_{0},v_{0}\right)$ denote the principal point coordinates. All of the above parameters are obtained through camera calibration.

**Table 2:** Measurement and ground truth data for monocular camera and MMW radar.

Actual Distance(m)	MMW Radar Measurement (m)	Camera Measurement (m)
5	5.04	5.02
10	10.07	10.1
15	15.1	15.39
20	20.13	20.47
25	25.17	25.77
30	30.24	31.2
35	35.33	36.7
40	40.37	42.1
45	45.44	47.4
50	50.51	53

**Table 3:** Average measurement error of millimeter-wave radar and monocular camera.

	MMW Radar	Camera
Mean Error(m)	0.237	1.22

**Table 4:** Mean RMSE of 15 datasets for three fusion algorithms, MMW radar, and monocular camera.

	Camera	Radar	IVW	EKF	AFEKF
Mean RMSE(m)	1.1524	0.2382	0.2397	0.2752	0.2131

## Data Availability

Due to the nature of this research, participants of this study did not agree for their data to be shared publicly and so supporting data are not available.

## Appendix A

**Table A1:** Explanatory table for parameter in Algorithm 1.

Symbol	Description
k	Discrete time index representing the k-th time step
$A_{k}$	State transition matrix of the target at time step k
$x_{0}$	Initial state vector of the target
H	Observation Matrix
$P_{0}$	Initial state estimation error covariance matrix
$P_{k}$	State estimation error covariance matrix during the iterative process
Q	Process noise covariance matrix
a,b,c	Parameters to be fitted in the distance error function
N	Total number of measurement data samples
i	Index of the sensor type in the fusion system
n	Number of sensor types
ε	Infinitesimal positive constant used for numerical stability

**Table A2:** Explanatory table for acronyms.

Acronym	Full Term
MMW	Millimeter-wave
AFEKF	Adaptive Fuzzy Extended Kalman Filter
EKF	Extended Kalman Filter
RMSE	Root Mean Square Error
IVW	Inverse Variance Weighting
ROI	Region of Interest
IOU	Intersection over Union
